# Identification of a genetically defined ultra-high-risk group in relapsed pediatric T-lymphoblastic leukemia

**DOI:** 10.1038/bcj.2017.3

**Published:** 2017-02-03

**Authors:** P Richter-Pechańska, J B Kunz, J Hof, M Zimmermann, T Rausch, O R Bandapalli, E Orlova, G Scapinello, J C Sagi, M Stanulla, M Schrappe, G Cario, R Kirschner-Schwabe, C Eckert, V Benes, J O Korbel, M U Muckenthaler, A E Kulozik

**Affiliations:** 1 Department of Pediatric Oncology, Hematology, and Immunology, University of Heidelberg, Heidelberg, Germany; 2 Molecular Medicine Partnership Unit, European Molecular Biology Laboratory, University of Heidelberg, Heidelberg, Germany; 3 German Consortium for Translational Cancer Research (DKTK), Heidelberg, Germany; 4 Department of Pediatric Oncology/Hematology, Charité Universitätsmedizin Berlin, Berlin, Germany; 5 Pediatric Hematology and Oncology, Hannover Medical School, Hannover, Germany; 6 European Molecular Biology Laboratory, University of Heidelberg, Heidelberg, Germany; 7 University of Padua, Padua, Italy; 8 Department of Pediatrics, University Hospital Schleswig-Holstein, Campus Kiel, Kiel, Germany

## Abstract

In the search for genes that define critical steps of relapse in pediatric T-cell acute lymphoblastic leukemia (T-ALL) and can serve as prognostic markers, we performed targeted sequencing of 313 leukemia-related genes in 214 patients: 67 samples collected at the time of relapse and 147 at initial diagnosis. As relapse-specific genetic events, we identified activating mutations in *NT5C2* (*P*=0.0001, Fisher's exact test), inactivation of *TP53* (*P*=0.0007, Fisher's exact test) and duplication of chr17:q11.2-24.3 (*P*=0.0068, Fisher's exact test) in 32/67 of T-ALL relapse samples. Alterations of *TP53* were frequently homozygous events, which significantly correlated with higher rates of copy number alterations in other genes compared with wild-type *TP53* (*P*=0.0004, Mann–Whitney's test). We subsequently focused on mutations with prognostic impact and identified genes governing DNA integrity (*TP53*, *n*=8; *USP7*, *n*=4; *MSH6*, *n*=4), having key roles in the RAS signaling pathway (*KRAS*, *NRAS*, *n*=8), as well as *IL7R* (*n*=4) and *CNOT3* (*n*=4) to be exclusively mutated in fatal relapses. These markers recognize 24/49 patients with a second event. In 17 of these patients with mostly refractory relapse and dire need for efficient treatment, we identified candidate targets for personalized therapy with p53 reactivating compounds, MEK inhibitors or JAK/STAT-inhibitors that may be incorporated in future treatment strategies.

## Introduction

Approximately 20% of children with precursor T-cell acute lymphoblastic leukemia (T-ALL) experience a relapse^1^ and only 20% of relapsed patients can be cured with current salvage protocols.^2^ Treatment after first relapse of T-ALL is uniform, using very intensive chemotherapy to induce second remission followed by allogeneic stem cell transplantation.^3, 4^ This treatment fails in the majority of patients,^2, 5^ indicating the need to identify patients who may benefit from experimental therapies. With the intensity of both frontline and relapse therapies, treatment-related mortality is a significant cause of treatment failure. Therefore, further intensification of chemotherapy is not an option resulting in the need for accurate prognostic markers and for identification of druggable targets.

Relapse evolves via selection of clones and acquisition of mutations.^6, 7^ Nevertheless, as no common biological determinants were identified to explain these events, our understanding of the mechanisms underlying relapse and treatment resistance is still limited. Activating mutations of the nucleotidase *NT5C2* were identified in approximately 20% of relapsed, but not primary T-ALL patients and confer resistance to chemotherapy *in vitro*.^6, 8^ *TP53* mutations have previously been reported to be acquired in relapse in up to 24% of patients and correlated with poor prognosis.^9, 10^ Although intensively investigated,^10, 11, 12, 13, 14, 15, 16, 17, 18, 19, 20^ clinically meaningful genetic markers for risk stratification or for targeted treatment could neither be established in primary nor in relapsed T-ALL.

To identify potential prognostic biomarkers in relapsed pediatric T-ALL and to define critical steps in disease progression and in resistance to treatment, we subjected a large cohort of 214 pediatric T-ALLs to targeted sequencing. We used the Haloplex target capture technique to analyze 313 leukemia-related genes in 147 samples collected at initial diagnosis and in 67 samples at the time of relapse. In addition to single-nucleotide variants (SNVs) and small insertions/deletions (InDels), we identified copy number alterations (CNAs) affecting target genes by analyzing coverage data.

## Materials and methods

### Patients' clinical characteristics

Altogether leukemic samples of 214 patients were analyzed: 67 relapse samples (REL) and 147 samples collected at initial diagnosis (INI). No matched primary and relapse samples were included in our study. Of the initial diagnosis patients, 31 were treated according to ALL-BFM 2000 and 116 patients according to AIEOP-BFM ALL 2009 protocol. All relapse patients were recruited from the ALL-REZ BFM 2002 trial. Clinical characteristics of the analyzed patients were compared with the remaining patients from the cohort (Supplementary Table 1). Except for white blood cell count, the distribution of patients' features was representative for the entire cohort. Enrichment for patients with high white blood cell counts is a likely consequence of selection for the samples with sufficient DNA amounts for the analyses performed here. Bone marrow or blood samples were enriched for mononuclear cells by Ficoll density gradient centrifugation. DNA was purified from mononuclear cells using the Gentra Puregene Cell Kit (Qiagen, Hilden, Germany). From one patient (PATNR: 82) with an isolated extramedullary relapse DNA was extracted from a lymph node. MRD (minimal residual disease) response was assessed as described before.^1, 17^ The study was approved by the institutional review boards of the Charité Universitätsmedizin Berlin and the Medical Faculty Heidelberg. Informed consent was obtained in accordance with the Declaration of Helsinki.

### Targeted deep sequencing

The Haloplex Target Enrichment Kit (Agilent, Santa Clara, CA, USA) covered 324 genes comprising 5964 regions (Supplementary Table 2). In all, 58 348 amplicons covered a total of 3.04 Mbp. Target genes were selected based on previously published studies.^6, 8, 20, 21, 22, 23, 24, 25^

A pilot study confirmed that reducing the reaction volume during library preparation resulted in a complexity of libraries equivalent to the standard reaction volume (Supplementary Results, Supplementary Figure 1). To save on input sample DNA and on costs, all subsequent reactions were performed in half a standard reaction volume. DNA was quantified using Qubit dsDNA BR Assay kit (Life Technologies, Darmstadt, Germany). Starting material was 112.5 ng of genomic DNA. The volume of all the reagents described in the manufacturer's instructions (Version D.5, May 2013) was reduced by half. Libraries were pooled in batches of 43 (1) or 44 (5) samples. Each batch was sequenced as 100 bp paired reads on one lane using an Illumina HiSeq 2000 instrument (Illumina, San Diego, CA, USA). VarScan^26^ was used to detect both SNVs and small insertions and deletions. Coverage profiles were used to identify copy number variations (for details see Supplementary Methods).

### Multiplex ligation-dependent probe amplification

The commercially available SALSA MLPA P383 T-ALL probe mix (MLPA (multiplex ligation-dependent probe amplification); MRC-Holland, Amsterdam, The Netherlands) and a custom-made probe set based on the SALSA MLPA P200-A1 probe mix (MRC-Holland; Supplementary Table 3) were used for the detection of specific copy number variations (Supplementary Methods).

### Low-coverage whole genome sequencing

Libraries for low-coverage WGS (whole genome sequencing) were prepared using NEBNext Ultra DNA Library Prep Kit for Illumina (New England Biolabs, Frankfurt am Main, Germany) from 100 ng of genomic DNA. Ten samples were pooled and sequenced on one Illumina HiSeq 2000 lane. Mean DNA sequence coverage was 3-fold (range 2–5-fold).

### Sanger sequencing

Sanger sequencing of *PTEN* exon 7 and of *NOTCH1* PEST, TAD, HD-C and HD-N domains was done as described before.^16, 18^

### Statistical analyses

Statistical analyses were performed using GraphPad Prism 6.0. Prognostic factors analyses were conducted using either the SAS program (SAS-PC, v. 9.1, SAS Institute Inc., Cary, NC, USA) or R^27^(Package: Survival;^28^ for details, see Supplementary Methods).

## Results

### Targeted NGS identifies SNVs/InDels and CNAs with high sensitivity

We designed a Haloplex Panel targeting leukemia-related genes to cover 5964 exons of 324 genes that we had compiled on the basis of previously published data sets.^6, 8, 20, 21, 22, 23, 24, 25^ Of the 324 genes, 313 were sufficiently covered to be analyzed in all 214 pediatric T-ALL patients. Average coverage of all the exons in 214 samples was 424 (median=417). Ninety-seven percent of the exons were covered more than 30-fold. To determine the accuracy of the Haloplex panel and our analytic setup for the detection of SNVs and InDels, we compared next-generation sequencing (NGS) data with conventional sequencing of the *NOTCH1* and *PTEN* genes in 144 patients. The sensitivity of the targeted NGS approach was 93% (67/72) and 94% (17/18) for *NOTCH1* and *PTEN* mutations, respectively (for details, see Supplementary Results).

In addition to the DNA sequence, targeted sequencing also delivers the read count distribution that can be used to estimate the copy number of a specific DNA fragment.^29, 30^ For regions covered by the panel, CNA detection by coverage data resulted in a sensitivity of 99% as validated by two independent methods: MLPA and low-coverage WGS (see Supplementary Results).

### SNVs and CNAs in leukemia-related genes are similarly common at the time of initial disease and at the time of relapse

**Figure 1:**
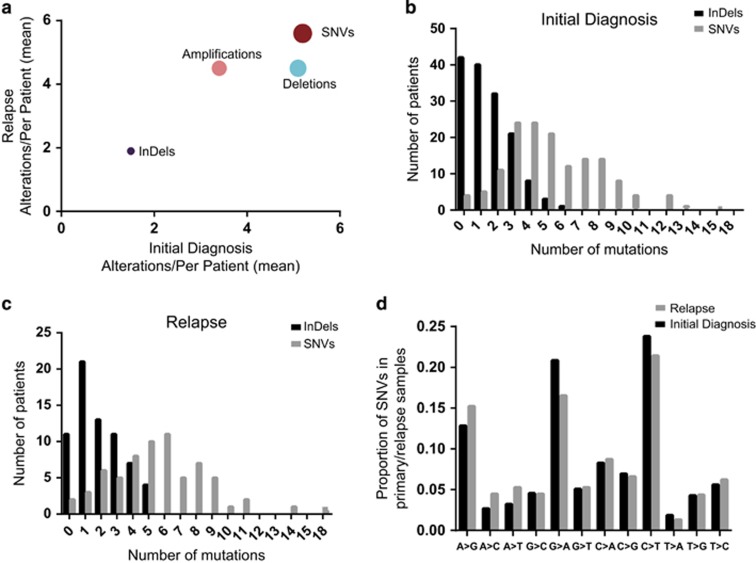
(**a**) Mean of SNVs, InDels, amplifications and deletions in initial diagnosis (*x*) plotted against relapse (*y*). Distribution of the number of SNVs (gray) and InDels (black) detected in the analyzed genes in the (**b**) initial diagnosis patients and in (**c**) relapse patients. (**d**) Frequencies of different types of missense mutations in primary and relapse samples.

**Figure 2:**
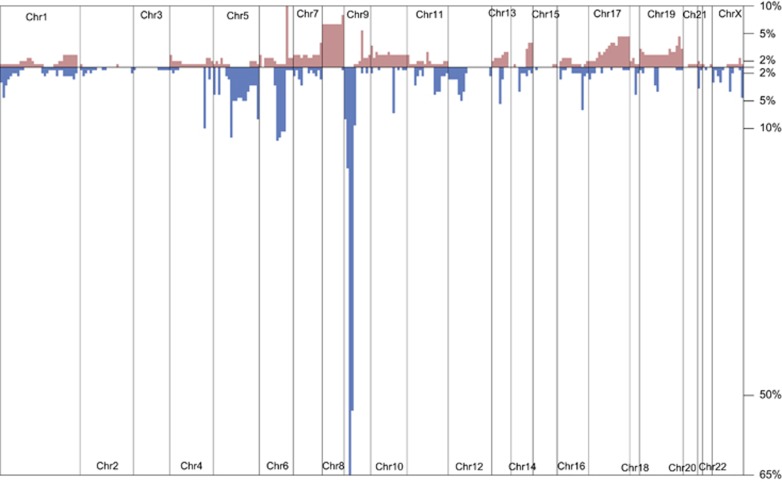
Frequencies of copy number alterations (red—mplifications; blue—deletions) detected in 202 T-ALL patients for whom coverage data from targeted sequencing were available. CNAs were plotted against their chromosomal position.

On average, gene panel sequencing detected seven SNVs or InDels per sample (in total 1496; Supplementary Table 5). We observed similar frequencies of SNVs in leukemia-related genes in the group of patients with initial disease and in the group of relapse patients (Figure 1a). InDels tended to be more frequent in relapse compared with primary leukemia (*P*=0.0618, Fisher's exact test; Figures 1b and c) suggesting that InDels may be a part of the mutational pattern induced by chemotherapy. Transitions constituted 61% of missense mutations with exchanges of C>T (22%) being the most common nonsynonymous mutations (Figure 1d), indicating that deamination of cytosine is a common mechanism of mutagenesis.^31^ Most of the mutations were found at allele frequencies of 35 to 65% (Supplementary Figure 2), indicating heterozygosity. Only few mutations were predicted to be homozygous, but the unknown blast content and subclonality of the samples do not allow for an unequivocal discrimination between homozygous and heterozygous events. We were able to perform CNA analyses in 202 of 214 samples (144 primary and 58 relapsed T-ALLs; Figure 2) and found that the average number of genes affected by CNAs in the leukemia-related genes of our panel does not differ between initial disease and relapse in pediatric T-ALL (INI: 8.6 vs REL: 9). As expected, CNAs most commonly affected chromosome 9 with *CDKN2A* and *CDKN2B* deletions detected in 66% and 55% of all patients, respectively.^32^ We observed recurrent deletions of regions on chr6:q14-15 (10%) and on chr5:q22-35 (up to 11% for *APC* INI: 20; REL: 3). Duplications of chromosome 8, including *MYC*, were the most common amplifications found in 7% of all patients (INI: 11; REL: 6; Figure 2, for details see Supplementary Results).

### Leukemogenic driver genes are characterized by high mutation densities

**Figure 3:**
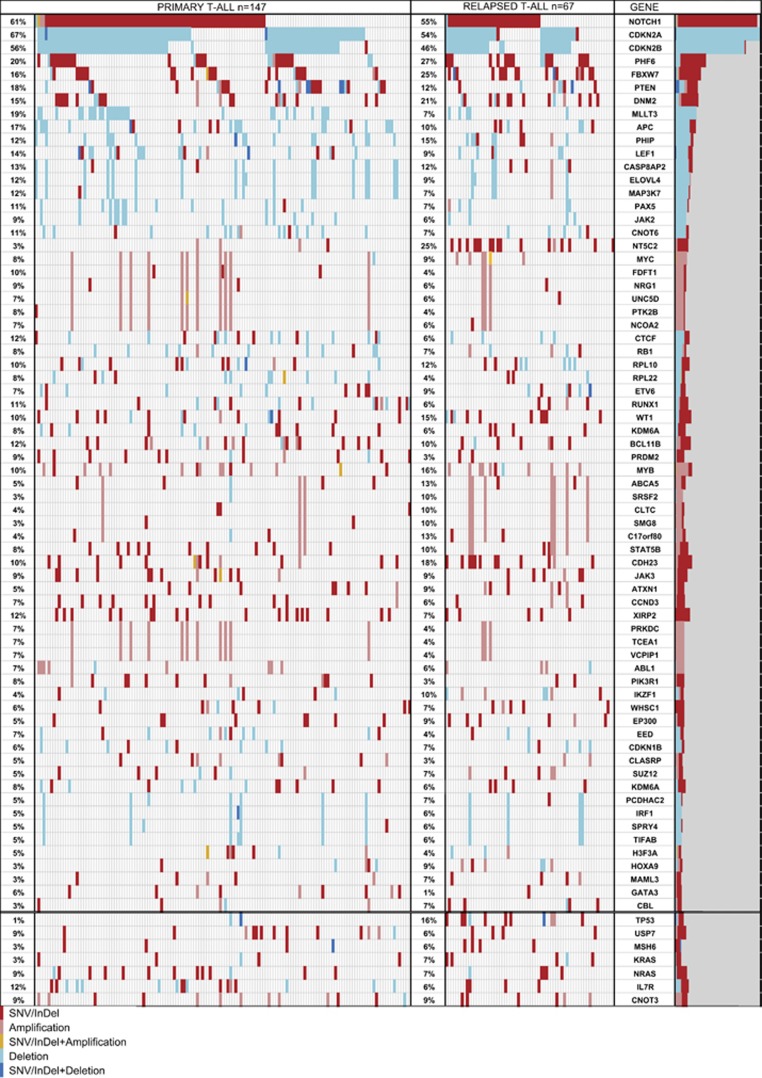
Comparison of the pattern of alterations in initial diagnosis patients (*n*=147) and in relapse (*n*=67) in the genes with high mutation density of SNVs/InDels (>1.9/Mbp; Supplementary Table 7) or/and high frequency of CNA (>5% of patients; data available for 202 patients: 59 REL and 144 INI). Frequencies refer to all mutations (SNVs/InDels+CNAs), alterations were sorted according to their frequency and position in the genome. Red—SNV/InDel; pink—amplification; orange—SNV/InDel+amplification; pale blue—deletion; dark blue—SNV/InDel+deletion.

We found known leukemia drivers to be mutated at the expected frequency^21, 33, 34^ (Figure 3). This includes among others *NOTCH1*, *PHF6*, *FBXW7* and *PTEN*, that had previously been found to be mutated at the frequency of approximately 50%, 16%, 20% and 17%, respectively. Other genes that were mutated in more than 10% of all patients were *DNM2*, *XIRP2* and *CDH23*. One hundred and eighty-two additional genes recurrently carried SNVs and InDels in less than 10% of patients.

**Figure 4:**
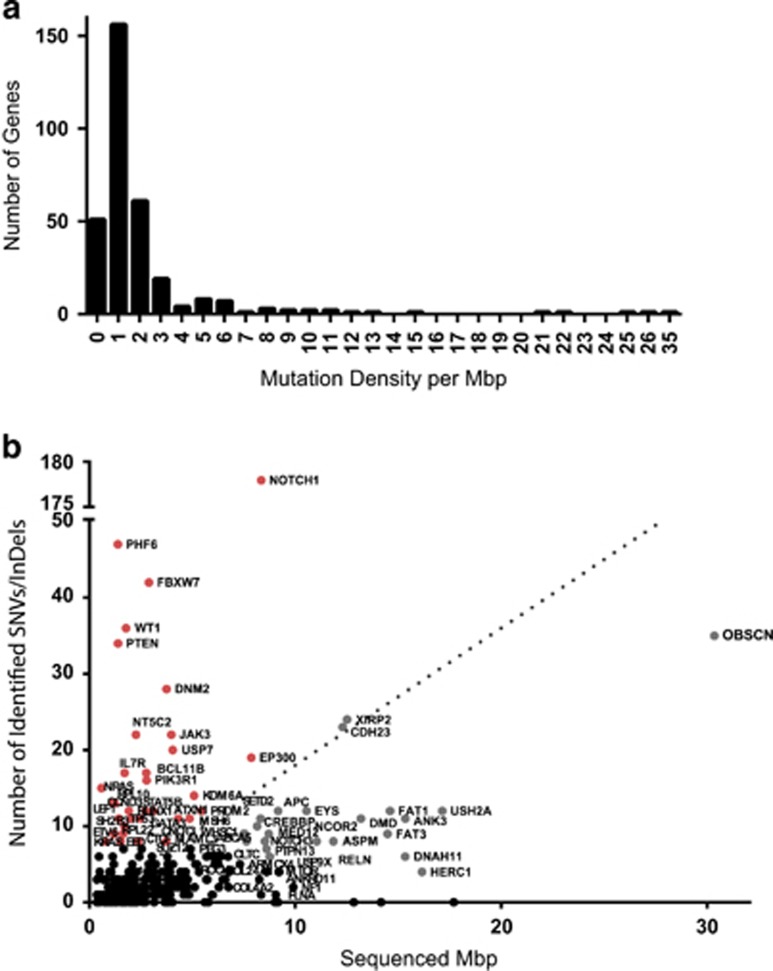
(**a**) Histogram of the distribution of mutation density in the analyzed genes (**b**) Mutation density shown as length of the targeted exons (Mpb) plotted against the absolute number of detected SNVs/InDels; red—known leukemia drivers or cancer-related genes; gray—genes that show low mutation density (<2), regardless of the high mutation frequency observed in some of them (for example, *OBSCN, XIRP2, CDH23*); black—genes that carry a low absolute number of mutations (*n*<10).

We next assessed which of the recurrently mutated genes likely represent drivers of leukemogenesis. The best way to distinguish driver genes from genes with randomly acquired (passenger) mutations would be through their pattern of mutations.^35^ However the low frequency of mutations in most of the analyzed genes precludes such an analysis. Therefore, we have calculated the mutation density for each gene by dividing the number of detected nonsynonymous SNVs and InDels by the length of the targeted exons per gene (mutations per Mbp; Supplementary Table 6). The mean mutation density for the regions covered by the panel was 1.8/Mbp. Most of the genes (77%, Figure 4) showed a mutation density below the mean. Genes with low mutation densities are likely to be randomly mutated and probably do not have a specific role in leukemogenesis, although some large genes in this category such as *OBSCN*, *EYS*, *DMD*, *FAT1*, *USH2A* and *ANK3* with a low mutation density between 0.7 and 1.2 were found to be mutated in a notable proportion of patients. This analysis confirmed the known leukemogenic role of genes with high mutation densities (>20/Mbp: *PHF6*, *NRAS*, *PTEN*, *NOTCH1*, *WT1*; >10/Mbp: *FBXW7*, *KRAS*, *IL7R*, *NT5C2*, *CCND3*, *RPL10*, *RPL22*). *XIRP2* and *CDH23*, both mutated in more than 20 patients but without an obvious link to leukemogenesis, had a much lower mutation density of 1.9/Mbp.

### T-ALL relapses are characterized by a limited number of specific alterations in leukemia-related target genes

As shown previously, *NT5C2* was the gene that most frequently carried relapse-specific mutations.^8, 36^ Twenty *NT5C2* SNVs were found in 16 (24%) of the 67 relapse samples and one *NT5C2* SNV was carried by leukemic cells of a patient at initial diagnosis (*P*=0.0001, Fisher's exact test). Thirteen out of 21 mutations detected in *NT5C2* were found in the hotspot positions R238, R367 and D407. These mutations cause a gain of function of this nucleotidase thus promoting the inactivation of nucleoside analogs that are used in the chemotherapy of T-ALL 8. Out of the 21 mutations detected in *NT5C2*, 15 (71%) were, as reported before,^6^ subclonal with an allele frequency (AF) of below 0.7 in proportion to the average of the AF in the major clone represented by mutations with the highest AF in the respective sample (Supplementary Table 5). This subclonality of *NT5C2* mutations suggests that these have been acquired later than the presumably clonal relapse-initiating event. Therefore, *NT5C2* mutations in T-ALL are likely not the relapse-initiating events. This observation is consistent with the lack of prognostic relevance of *NT5C2* mutations in relapsed T-ALL (see below).

*TP53* was the second most common gene to be mutated in a relapse-specific fashion. Only 2/147 samples at initial disease carried alterations of *TP53*, whereas we detected *TP53* SNVs (7 missense, 1 stopgain, 1 frameshift deletion) or large deletions (2) in 9/67 samples of relapse patients (*P*=0.0007, Fisher's exact test). Mutations of *TP53* detected at relapse were individually non-recurrent and affected either the transactivation or DNA-binding domains. SIFT,^37^ MutationTaster^38^ and PolyPhen-2^39^ concordantly predicted these mutations to be damaging for p53 function (Supplementary Table 5). None of the *TP53* mutations were detected at the known activating hotspots.^40^ Patients who carried SNVs or deletions of *TP53* showed higher mutation rates in other genes compared with those with wild-type *TP53* (31 vs 14 alterations per patient, *P*=0.0002, Mann–Whitney's test). This difference was particularly notable in the frequencies of CNAs (mut*TP53*: 24 vs wt*TP53*: 8 per patient, *P*=0.0004, Mann–Whitney's test), whereas the difference in SNVs and InDels was less pronounced (mut*TP53*: 8 vs wt*TP53*: 6 per patient, *P*=0.0036, Mann–Whitney's test). These findings suggest that disruption of *TP53* function in T-ALL results in an accumulation of somatic duplications and deletions.

In addition, we identified amplifications of a region on chromosome 17 q11.2-24.3, represented in our panel by *STAT5B*, *STAT5A*, *STAT3*, *DHX8*, *SMG8*, *CLTC*, *ABCA5*, *C17orf80*, to be significantly enriched in relapse (REL 7/58 vs INI 3/144, *P*=0.0068, Fisher's exact test). Because targeted sequencing does not allow to define exact borders of CNAs, three of the samples that carry amplifications of large regions of chromosome 17 were analyzed by low-coverage WGS. We thus resolved the amplified region to chr17: q21.23-q25.3 in two samples (PATNR: 20; 22) and to chr17: p11.2-q25.3 in the third sample (PATNR: 50). There was no significant association of amplifications of chromosome 17 with prognosis in relapse. However, this region is enriched in genes of the JAK/STAT pathway that has been implicated in leukemogenesis^25^ and in the *ABCA* gene family that has been implicated in drug resistance.^41^ As these two groups of genes may offer options for targeted treatment,^41, 42, 43^ it will be important to identify this amplification in future studies.

### Fatal outcome in relapsed T-ALL is heralded by the mutational profile

Clinical follow-up data were available for 66 of the 67 relapse patients. Forty-nine of these patients (74%) suffered an event (second relapse, death or secondary malignancy), whereas 17 (26%) could be salvaged by relapse therapy.

*TP53* mutations were most highly predictive of a second event. For one out of nine patients with alterations in *TP53* in leukemic cells, clinical data were not available. All other patients who carried a total of nine *TP53* mutations and/or deletions died within 9 months after first relapse, whereas 17 of 58 patients without *TP53* mutation (29%) survived (*P*=0.001, log-rank test). Five of ten *TP53* mutations detected in our entire cohort had an AF equal or above 74%. Such high allele frequencies were found in only 106/1507 of all SNVs and InDels in our analysis (Supplementary Figure 2), suggesting that *TP53* mutations were more frequently homozygous events than mutations in the other analyzed genes. Two were a consequence of loss of heterozygosity through a deletion of the second allele (patients GW15, PATNR: 14). In three more samples, no CNAs were detected but all SNVs and single-nucleotide polymorphisms in *TP53* were homozygous, indicating that loss of heterozygosity had occurred via uniparental disomy. Therefore, there is a strong selective pressure that results in the acquisition of homozygous *TP53* inactivation in relapsed pediatric T-ALL.

By contrast, *NT5C2* mutations, highly enriched in relapse, had no prognostic impact in our series. Although patients who harbored *NT5C2* mutations had relapsed earlier (*P*=0.01, Fisher's exact test, Supplementary Table 7), the frequency of *NT5C2* mutations did not differ between patients who suffered a second event (12/49) and patients who were salvaged by second line treatment (4/17; *P*=1, Fisher's exact test).

We next focused on mutations in those genes that were not significantly enriched in relapse, but when found at relapse occurred only in samples from patients who suffered a second event and death. These were *USP7*, *MSH6*, *KRAS*, *NRAS*, *CNOT3* and *IL7R*.

**Figure 5:**
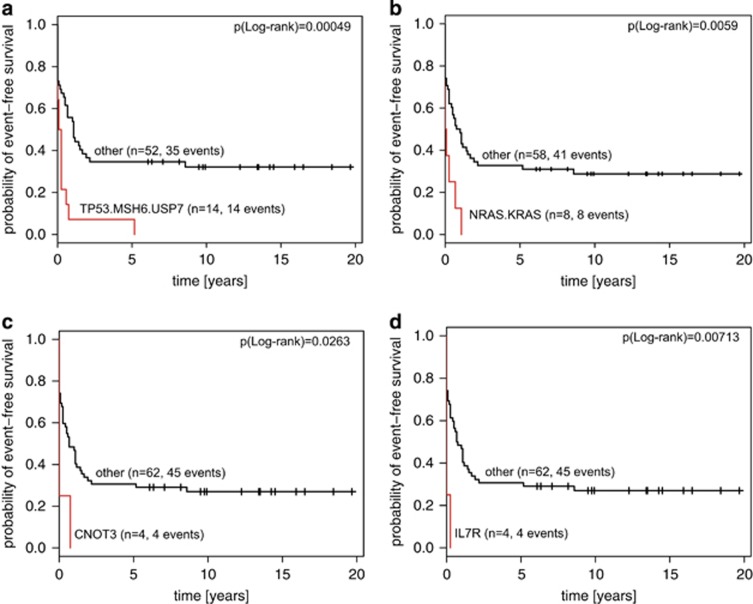
Kaplan–Meier plots of event-free survival in the group of 66 relapse patients. (**a**) Patients who carry SNV/InDel or deletions in *TP53/MSH6/USP7* vs other. (**b**) Patients who carry mutations in *KRAS* or *NRAS* vs other. (**c**) Patients who carry SNVs in *CNOT3* vs other. (**d**) Patients who carry mutations in *IL7R* vs other.

Mutations in *USP7*, that deubiquitinates p53 preventing its degradation and enhancing p53-dependent transcription regulation, cell growth repression and apoptosis,^44^ were found in four relapse samples. Three patients carried a frameshift insertion of *USP7* and one carried a missense variant predicted to be damaging for the structure of the protein (Supplementary Table 5). All except one were located in the catalytic domain of *USP7*. Another gene that is responsible for maintenance of genetic stability and tumor suppression and which was mutated exclusively in relapse patients with fatal outcome was the DNA mismatch repair gene *MSH6*. It recognizes mismatched nucleotides before their repair. Six *MSH6* mutations were found in four relapse samples. All samples carried at least one mutation in the sequence coding for the conserved MutS domain. We detected three *MSH6* mutations leading to a stop codon, two InDels and one missense mutation concordantly predicted to be deleterious by SIFT,^37^ Polyphen^39^ and MutationTaster.^38^ Together, mutations in *TP53*, *USP7* and *MSH6* define a group of fatal relapsed leukemias predicted to be defective in surveillance of DNA integrity (*P*=0.00049, log-rank test, Figure 5a).

The Ras/Raf/MEK/ERK pathway has a crucial role in the transmission of proliferative signals from membrane-bound receptors. Ras mutations were found in eight relapsed patients, while no mutation of the downstream target of Ras signaling, *BRAF*, was detected in our cohort. Seven out of eight *NRAS* and *KRAS* mutations were detected at known activating hotspots (G12, A59, Q61 and A146) or in their close proximity (V14). All relapsed patients with *KRAS* or *NRAS* mutations in leukemic samples died, six out of eight within 3 months after relapse, indicating that these mutations are associated with treatment resistance (*P*=0.0059, log-rank test, Figure 5b).

*CNOT3* and *IL7R* are similarly associated with early treatment failure after relapse (Figures 5c and d). *CNOT3* was previously identified as a tumor suppressor in 8% of adult T-ALLs.^23^ Out of four *CNOT3* mutations, three were found in a region coding for the N-terminal coiled-coil domain and one directly on the border with a linker region (*CNOT3-*M). One of the mutations was an exchange of Arginine 57 in a hotspot position previously reported to interfere with the tumor suppressor function of *CNOT3*.^23^ One of the mutations is a frameshift insertion and the effect of the two remaining mutations is damaging for the protein structure based on predictive algorithms. All four patients harboring *CNOT3* mutations at relapse died, three of them within 1 month after diagnosis. The same applies to patients with *IL7R* mutations. All *IL7R* mutations were found at the hotspot positions coding for Leucine 242 and 243, three out of four being nonframeshift insertions introducing an unpaired cysteine and inducing ligand-independent constitutive hyperactivation of IL7R-mediated signal transduction.^42, 45^

Mutations of genes governing DNA integrity (*TP53*, *USP7*, *MSH6*; *n*=15 patients) and in the key players of the RAS signaling pathway (*KRAS*, *NRAS*; *n*=8 patients) combined with alterations of *IL7R* (*n*=4) and *CNOT3* (*n*=4) represent a prognostic signature that recognizes 24/49 (49%) relapse patients with fatal outcome. Notably, out of those 23 patients for whom clinical data are available, 13 were resistant to treatment and did not reach second remission. Six more patients suffered from an early second relapse and died before proceeding to stem cell transplantation. Of the four patients who proceeded to stem cell transplantation, three died after relapse and one experienced a secondary malignancy. In comparison, only nine of 43 patients without high-risk mutations failed to reach second remission (*P*=0.0058, Fisher's exact test).

## Discussion

### Recurrent SNVs and InDels

We compared mutational patterns of point mutations and CNAs in a large group of 147 primary and 67 relapsed T-ALL patients by targeted sequencing of more than 300 genes. The distribution of the most common SNVs/InDels detected in our cohort is in accordance with published data, reproducing the frequent association of T-ALL with mutations in *NOTCH1*, *FBXW7*, *PHF6*, *PTEN, IL7R*, *WT1* and others^34, 46^ (Figure 3). Although we did not identify novel common driver genes that were mutated in more than 10% of patients, 140 of 147 primary T-ALL samples carried at least one mutation in a gene that was mutated in 2 to 10% of all patients. We hypothesize that at least some of those genes that are recurrently mutated at low frequency may contribute to leukemogenesis and that these rare genetic alterations contribute considerably to the genetic diversity of T-ALL.

In our previous whole-exome sequencing analysis of matched samples from primary and relapsed T-ALL, we observed that the number of detected SNVs and InDels between initial diagnosis and relapse doubled,^6^ while the numbers detected by targeted sequencing as performed here were not significantly higher in relapse. This is likely explained by exome sequencing identifying both, the accumulation of driver and passenger mutations. By contrast, leukemia-related mutations detected by targeted sequencing do not show a major change in number during the evolution to relapse.

### Panel sequencing allows for robust detection of CNAs

The feasibility of targeted NGS for the detection of CNAs was shown by validation by two independent methods: MLPA and WGS. Moreover, genome-wide copy number analyses using Affymetrix single-nucleotide polymorphism arrays in 73 (ref. 32) and 50 (ref. 47) T-ALL patients show a high concordance in terms of frequencies of deletions and amplifications with those found in genes covered by our analyses. Within the group of selected regions contained in the Haloplex panel, genes were more frequently affected by duplications or deletions than by point mutations and InDels (mean number of SNVs/InDels per gene: 7, median: 7; CNA: mean: 8.6, median: 5). In 31 of 202 (15%) samples for which CNA data were available we observed large regions to be affected, possibly affecting entire chromosomes. Assuming that the proportion of genes affected by CNA within our panel is representative for the entire genome, we estimate that in average between two and three percent of the genome is altered by copy number changes in pediatric T-ALL.

### *TP53* and *Ras* genes confer a dismal prognosis in relapsed T-ALL

Mutations detected in *NT5C2* and in *TP53* were significantly enriched in relapse. Activating *NT5C2* mutations were previously reported to be relapse-specific^36^ and to drive resistance against 6-mercaptopurine and 6-thioguanine in relapsed ALLs.^8^ Twelve out of 16 *NT5C2*-positive relapse patients carried at least one mutation of the known activating hotspot. In the four remaining leukemic samples, missense exchanges at positions R34Q, R195Q and T489M and nonframeshift deletion of four amino acids at positions 396-400 were detected.

*NT5C2* mutations are associated with early first relapse, indicating that the acquisition of *NT5C2* mutations is driven by selective pressure. Induction failure after relapse is not predicted by *NT5C2* mutations, most likely because derivatives of 6-mercaptopurine are not a component of induction treatment after relapse.

Alterations of *TP53* were reported to be gained at relapse in 54% of 23 ALL samples and found to predict chemotherapy resistance and poor outcome in first relapse of childhood B-cell-precursor ALL.^10^ In T-ALL, *TP53* mutations were previously detected in first relapse in 12 of 51 patients (24%).^9^ Patients with *TP53* mutations in leukemic samples experienced a shorter duration of survival and were significantly less likely to have achieved a complete second remission than patients without *TP53* mutations.^9^ Our results confirm and extend these earlier findings. We find *TP53* to be mutated in a relapse-specific manner with high allele frequencies, indicating homozygous events. Moreover, *TP53* mutants were characterized by a higher overall mutation rate in comparison with *TP53* wild-type patients, which was particularly evident by the accumulation of CNAs. It has been demonstrated that *TP53* mutations result in the accumulation of genomic rearrangements in several cancer entities.^48, 49, 50^ However, we cannot formally exclude that mutations in *TP53* are not a cause but the consequence of genomic instability.

T-ALLs that showed such genetic instability as a result of *TP53* mutation failed treatment within 9 months after relapse. When we combine *TP53* alterations with recurrent mutations in *MSH6* and *USP7*, which also interfere with the regulation of DNA surveillance, we can define a group of approximately 20% of patients with invariably fatal outcome in first relapse (15/67).

Mutations in *NRAS* and *KRAS* recognize eight relapsed patients with fatal outcome, although neither of these mutations had significant prognostic impact in primary disease (*P*=0.15 and 0.49, respectively, Gray's test). Deregulation of the Ras/Raf/MEK pathway has repeatedly been implicated in resistance to chemotherapy.^19, 51^ This may be a result of constitutive activation of its direct components (*NRAS*, *KRAS* and *BRAF*), upstream receptors (*EGFR*, *FLT3*) or chimeric chromosomal translocations (BCR–ABL, TEL–PDGFR). Targeting this pathway may be a novel therapeutic approach for drug resistant leukemia, which is often cross-resistant to multiple chemotherapeutic drugs.^52^ With relapsed T-ALL being a rare entity, it is fortunate that numerous compounds targeting the Ras/Raf/MEK pathway, such as the small molecules trametinib or selumetinib, have been tested in other conditions and could potentially be repurposed for the treatment of T-ALL.

In addition, mutations in *CNOT3* and *IL7R* are exclusively found in patients experiencing treatment failure after first relapse. Although a correlation of *IL7R* mutations with prognosis in primary T-ALL could not be established,^43^ *IL7R* mutations in relapse were correlated with very short survival (*P*=0.007, log-rank test). These findings have potential therapeutic implications as it was shown that T-ALL cells harboring *IL7R* mutations are sensitive to JAK–STAT pathway inhibitors.^42^

Although the low frequency of these mutations individually precludes a separate analysis as prognostic biomarkers, a combination of these functionally related genes identifies 49% (24 of 49) of all patients that fail relapse treatment, mainly as a result of nonresponse. Twelve of these 24 patients would have been candidates for targeted treatment with either JAK–STAT or MEK inhibitors. Six patients that carry missense mutations in *TP53* could potentially benefit from agents that restore the active conformation of mutant p53 and induce p53-dependent suppression of tumor cell growth as currently being tested in other cancer indications.^53, 54^ We are currently developing xenograft models of genetically well-defined leukemias that will allow correlating the mutational profile with drug sensitivity and will thus enable us to directly test the potential to use the signature reported here to design new targeted treatment strategies.

In reverse, the absence of mutations in *TP53*, *MSH6*, *USP7*, *NRAS*, *KRAS*, *CNOT3* and *IL7R* defines a group among T-ALL relapse patients with a 40% chance of survival with conventional relapse treatment (Supplementary Figure 3).

## Supplementary Material

Supplementary Results
