# Supplementary material for: Identification of a genetically defined ultra-high-risk group in relapsed pediatric T-lymphoblastic leukemia
Source: Blood Cancer J. 2017 Feb 3;7(2):e523–. doi: 10.1038/bcj.2017.3 (PMC5386337; doi:10.1038/bcj.2017.3)
Supplement: Supplementary Results [file bcj20173x1.docx]

## Supplementary Results

### Half-reaction pilot study

In order to reduce the amount of input DNA and costs of the Haloplex reagents we have performed a pilot study in which we compared libraries that had been prepared in half the standard reaction volume with libraries prepared according to the manufacturer’s instructions. For 8 samples libraries were prepared in parallel using the full and the half volume of the reagents described in the protocol. DNA input was respectively reduced by half.

There was no statistically significant difference in the average coverage of target regions (p=0.5, t-test, Suppl. Fig. 1). No difference was observed in the number of regions with coverage below 30 reads (p=0.68) or those that were not covered (p=0.16). All mutations that were detected in the standard reactions were also detected in the half reaction libraries. Reducing the volume during library preparation did not result in the detection of any new, artefactual mutations. Moreover, no statistically significant difference was observed in the allele frequency between the both (p=0.22). We conclude that both DNA input and reagents for library preparation can be reduced by 50% without any loss in sensitivity and specificity with regard to the detection of mutations. All further library preparations were performed in half the standard reaction volume.

### SNVs/InDels validated by Sanger sequencing

Sanger sequencing data of *NOTCH1* (PEST, HD-N, HD-C and TAD domains) and *PTEN* (exon 7) were available for 144 samples. Seventy-two *NOTCH1* mutations were found using conventional sequencing. Out of these, 67 were also identified by Haloplex resulting in a sensitivity of 93%. Four of the five missed mutations were InDels. In the fifth sample, the region of interest was poorly covered by Haloplex (14 reads) and the required minimum of at least five variant reads was not reached. Out of 18 *PTEN* exon seven InDels identified by Sanger sequencing, 17 (94%) were called by Varscan in Haloplex data. Additional four subclonal mutations that were not identified by conventional sequencing were found by targeted sequencing in exon 7. All four have allele frequency of less than 10%. These results demonstrate that panel sequencing by Haloplex largely recapitulates results from Sanger sequencing and is superior to Sanger sequencing in the detection of subclonal mutations.

### Validation of CNA detection

We calculated a normalized coverage of targeted sequencing for each region and validated these data by comparison with those obtained by MLPA for 13 overlapping regions covering 14 genes (Suppl. Tab. 4) in 182 patients (n=2 366 regions). Altogether MLPA identified 406 CNAs (47 amplifications and 359 deletions) in 182 samples. Out of the 359 deletions detected by MLPA, 36 were predicted to be undetectable by coverage analysis: eight were the result of poor probe binding caused by either SNV or InDels and eleven were subclonal. In nine cases a single exon was observed to be deleted by MLPA and the threshold set for coverage analysis required at least 3 adjacent exons to define a deletion. In eight further samples the ratio observed in MLPA analysis was very close or slightly above the threshold of 0.7.

Out of the 370 remaining CNAs, 365 (99%) could be identified using the coverage data from targeted NGS. Setting MLPA results as a standard, the sensitivity of Haloplex sequencing was 100% for amplifications (47/47); 100% for biallelic deletions (226/226) and 95% for monoallelic deletions (92/97). Out of 2 366 analyses we identified nine false positive results, which resulted in a specificity of 99% (Suppl. Tab. 4). At the same time read depth analysis detected 2 additional amplifications and 9 deletions that were not recognized by MLPA. Five of these were deletions of *CDKN2B*, which was covered by two regions in Haloplex analysis and only one MLPA probe, explaining the higher sensitivity of coverage analysis. Because we did not use a third method of CNA detection we cannot ascertain that these were true positives, but we can conclude that the sensitivity of Haloplex sequencing for detection of specific CNAs closely equals that of MLPA.

Additionally, we have validated amplifications found recurrently in six genes (*ABCA5*, *AKT1*, *CNOT3*, *MYC*, *PTK2B* and *STAT5B*) and deletions detected in three genes (*CNOT6*, *CTCF*, and *PDGFRB*) in 54 samples from the relapsed cohort. All eleven duplications and eleven deletions detected by MLPA were as well identified in coverage data (Suppl. Tab. 4b). In eleven additional cases we detected an amplification in coverage data, while results of the MLPA were difficult to interpret (UD), but consistent with NGS. Additionally targeted sequencing identified one amplification of *ABCA5* that was not found by MLPA analysis.

Low coverage WGS was used as second independent method to validate CNA results obtained from Haloplex coverage data in a subset of 7 samples. Forty-four deletions or amplifications spanning at least 1000 bp in four relapse and three primary samples were detected by Haloplex read depth analysis. All were confirmed by low coverage WGS (for exemplary read depth plots comparison see Suppl. Fig. 4).

### CNAs are common events in T-ALL

We have found deletions of a region on chromosome 5q (14.3 - 35.3) including the genes *MSH3*, *MEF2C*, *IRF1*, *TIFAB*, *ANKHD1*, *EIF4EBP3*, *PCDHAC2*, *SPRY4*, *PDGFRB*, *ODZ2*, *WWC1*, *DOCK2* and *CNOT6* in up to 11 of 214 patients. Within this large region, we found the tumor suppressor gene *APC* (located on 5q21-q22) to be inactivated in 11% of patients from our cohort. APC is a negative regulator of the Wnt/β-catenin pathway that is implicated in the regulation of hematopoietic stem proliferation and differentiation. Deletions of 5q are the most frequent chromosome anomaly in myelodysplastic syndrome and acute myeloid leukemia and were sporadically reported in acute lymphoid leukemias ^64^. The cluster between 5q13 and 5q33 contains genes that are involved in the regulation of hematopoiesis, including cytokines and their receptors, cell cycle regulators, transcription factors, and signaling mediators ^65^. One further gene on chr:5q33 that has been linked to leukemogenesis ^66^ is *RPS14*, coding for a ribosomal protein. Taken together, mutations or deletions in *RPS14*, *RPL5*, *RPL10*, *RPL11* and *RPL22* are found in 51 of 214 samples in our cohort, confirming that T-ALL frequently is associated with ribosomal defects ^67^.

Amplifications were less common than deletions, as previously reported ^32^, with an average of 3.7 amplifications per sample. Most frequent were amplifications of *MYB* found in 10% and of *MYC* in 8% of patients as previously reported^68-70^. Similarly, *PTK2B*, a gene that has been found to be deregulated by fusion in Philadelphia-like BCP-ALL and that is potentially targetable by tyrosine kinase inhibitors, is duplicated in 8% of primary T-ALL. *MYC and PTK2B* were frequently part of a larger gain, possibly a trisomy of chromosome 8, represented in our panel by the genes *FDFT1*, *PTK2B*, *NRG1*, *UNC5D*, *PRKDC*, *TCEA1*, *VCPIP1*, and *NCOA2*. Further studies are required to determine the contribution of single genes in this commonly amplified region to leukemogenesis in T-ALL.
