# Supplementary material for: Identification of a genetically defined ultra-high-risk group in relapsed pediatric T-lymphoblastic leukemia
Source: Blood Cancer J. 2017 Feb 3;7(2):e523–. doi: 10.1038/bcj.2017.3 (PMC5386337; doi:10.1038/bcj.2017.3)
Supplement: Supplementary Methods [file bcj20173x2.docx]

## SUPPLEMENTARY INFORMATION

## Supplementary Methods

### Single Nucleotide Variants and InDels Detection

Cutadapt ^55^ was used to remove Haloplex-specific adapter sequences from fastq files. Trimmomatic ^56^ was used to filter and trim low quality bases and to crop the first 5bp that can be reference-biased due to the Haloplex restriction enzyme footprint. All trimmed reads shorter than 36 bp were discarded. Preprocessed fastq files were mapped against the human reference genome hg19 (hg19, GRCh37 Genome Reference Consortium Human Reference 37 (GCA_000001405)) using bwa (Burrows-Wheeler Aligner) mem algorithm ^57^. Bam files were sorted and indexed by samtools ^58^. Identified variants were functionally annotated using ANNOVAR ^59^ tool with the corresponding nucleotide exchange, and then compared with those listed in dbSNP v138 (http://www.ncbi.nlm.nih.gov/snp/) and in the latest release of the 1000 Genomes Project (August, 2014; http://1000genomes.org). Annotations include as well GERP conservation scores ^60^ and indications whether the variant is located in a segmental duplication region (SegDup). Predictions of the functional impact of amino acid exchanges on the structure and function of the respective protein were computed using SIFT ^37^, MutationTaster^38^ and PolyPhen-2^39^. All SNVs were filtered for nonsynonymous, stopgain/stoploss, frameshift, and nonframeshift requiring at least five supporting reads for a called variant. Variants that were present in either 1000 Genome Project release 2014 with a frequency of more than 1% or in dbsnp138 were subtracted (except for *KRAS* and *NRAS* hotspots). We have selected for the variants with AF of at least 10%.

For the discrimination of somatic from germ line mutations, we used several filters: First, known SNPs (dbSNP, 1000 gp) were subtracted; second, two controls were sequenced along with the patients’ samples. Each of the controls comprised of 10 pooled non-leukemic samples. Variants of a frequency higher than 5% (present in at least one of the samples in a pool as a heterozygous variant) were excluded from the analyses. Nevertheless we are aware of the fact that we cannot in every instance safely distinguish between somatic and germ line events in the absence of a matched germ line sample.

All predicted mutations were visually inspected by integrated genome viewer (IGV) ^61^ in order to decrease the false positive rate. Identified sequencing artifacts were excluded from further analyses.

### Copy Number Alterations Detection

Average coverage of genomic intervals defined as regions in the bed file was annotated using DELLY Cov ^62^. Each region usually corresponded to one exon. To minimize the effect of uneven representation of samples within one batch, for each sample the ratio of the reads per sample and the total number of reads per batch was calculated. Read count for every region in a sample was then divided by this ratio. Regions with median coverage lower than 100 reads as well as those showing high variability were excluded from the analyses. As a measure of variability of coverage we used the difference between the quantiles 90 and 10 for read depth in the covered regions, normalized by the median coverage. Samples with Q90-Q10/Median of > 1.7 were not amenable to further analysis.

For CNA detection in chromosome X average region coverage for female patients was divided by two. To identify CNA at individual regions a sample normalized average read count for each region was divided by the median read count of the batch in that region. For *CDKN2A* and *CDKN2B*, known to be frequently deleted in T-ALL, instead of the median read count of the batch in that region, a median coverage of 2 non-leukemic controls served to calculate the ratio. Genes with three or more adjacent exons showing read depths of 30% below the median value were considered deleted. Copy number gain was assigned to those genes for which the majority of the exons showed read depth increase of at least 25%.

### MLPA

The chromosomal areas and target genes are: *STIL*-*TAL1* (1p33), *LEF1* (4q25), *CASP8AP2* (6q15), *MYB* (6q23.3), *EZH2* (7q36.1), *CDKN2A*/B+*MTAP*+*MLLT3* (9p21.3), *NUP214*-*ABL1* (9q34.1), *PTEN* (10q23.31), *LMO1* (11p15.4), *LMO2* (11p13), *NF1*+*SUZ12* (17q11.2), *PTPN2* (18p11.21), *PHF6* (Xq26.2). Seventy ng of starting material were processed according to the manufacturer's protocol (version MDP-v003). Biallelic deletions were defined with at least 50% reduction of relative peak area of the amplification product of that probe, monoallelic deletions with a reduction of 30-50%. A threshold for amplifications was set at a 25% increase of the probe signal.

### Statistical Analyses

Event-free survival (EFS) was defined as the time from diagnosis to the date of last follow up in complete remission or first event. Events were resistance to therapy (non-response), relapse, secondary malignancy, or death from any cause. Failure to achieve remission due to early death or non-response was considered as events at time zero. Survival was defined as the time from diagnosis until death from any cause or last follow-up. Analyses were performed with the R-package “Survival”. The Kaplan-Meier method was used to estimate survival rates, differences were compared with the two-sided log rank test. Cox’s proportional hazards model was used for uni- and multivariate analyses. Cumulative incidence (CI) functions for competing events were constructed by the method of Kalbfleisch and Prentice, and were compared with the Gray´s test using the cmprsk package for R ^27, 63^. Results are presented as estimated probability of 5-year EFS (pEFS). Differences in the distribution of individual parameters among patient subsets were analyzed using Chi-Square-Test or Fisher’s exact test for categorized variables and the t-test test for continuous variables.
