# Supplementary figures and images for: Identification of a genetically defined ultra-high-risk group in relapsed pediatric T-lymphoblastic leukemia
Source: Blood Cancer J. 2017 Feb 3;7(2):e523–. doi: 10.1038/bcj.2017.3 (PMC5386337; doi:10.1038/bcj.2017.3)

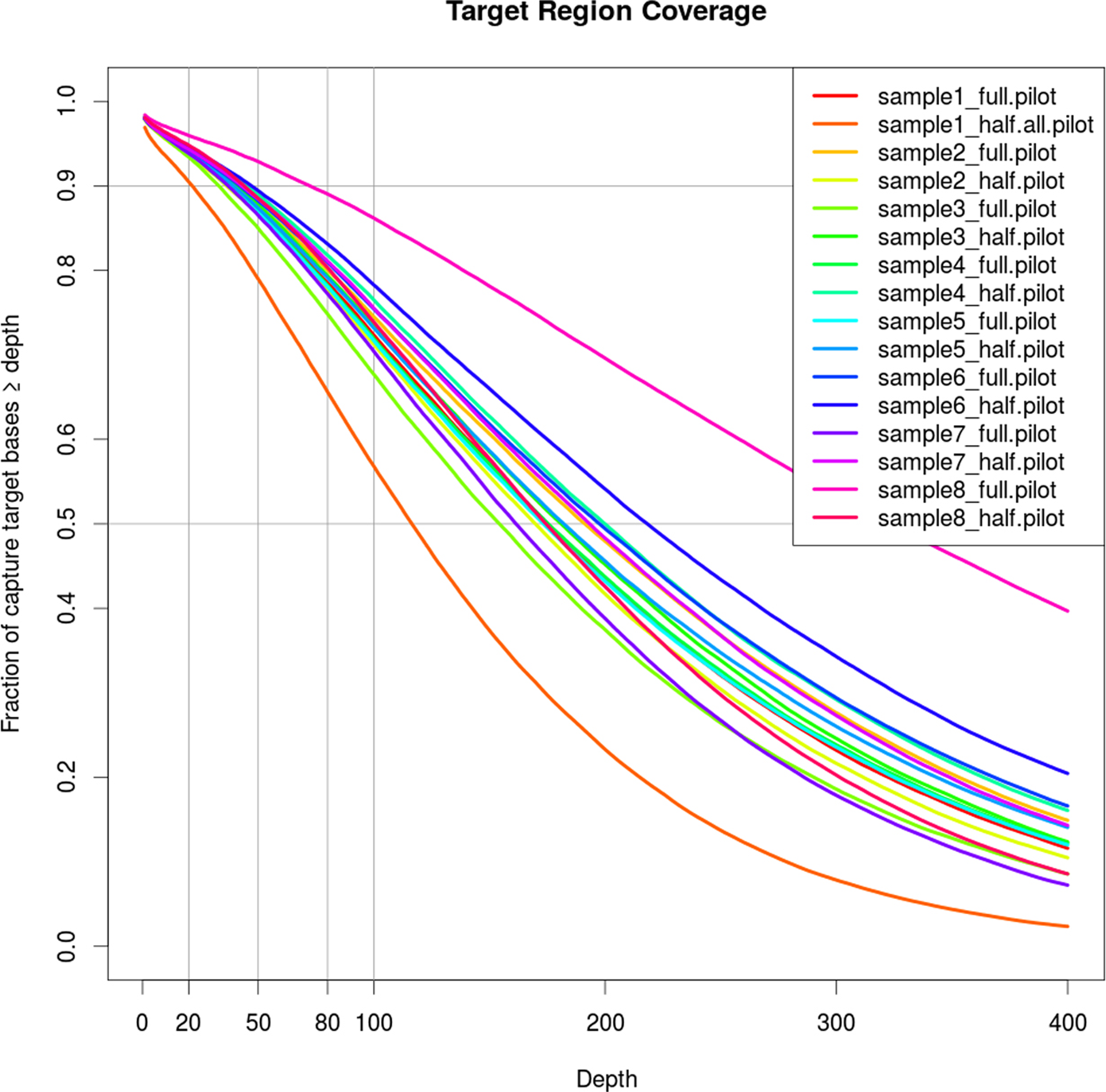

Supplement: Supplementary Figure 1 [file bcj20173x3.tif]

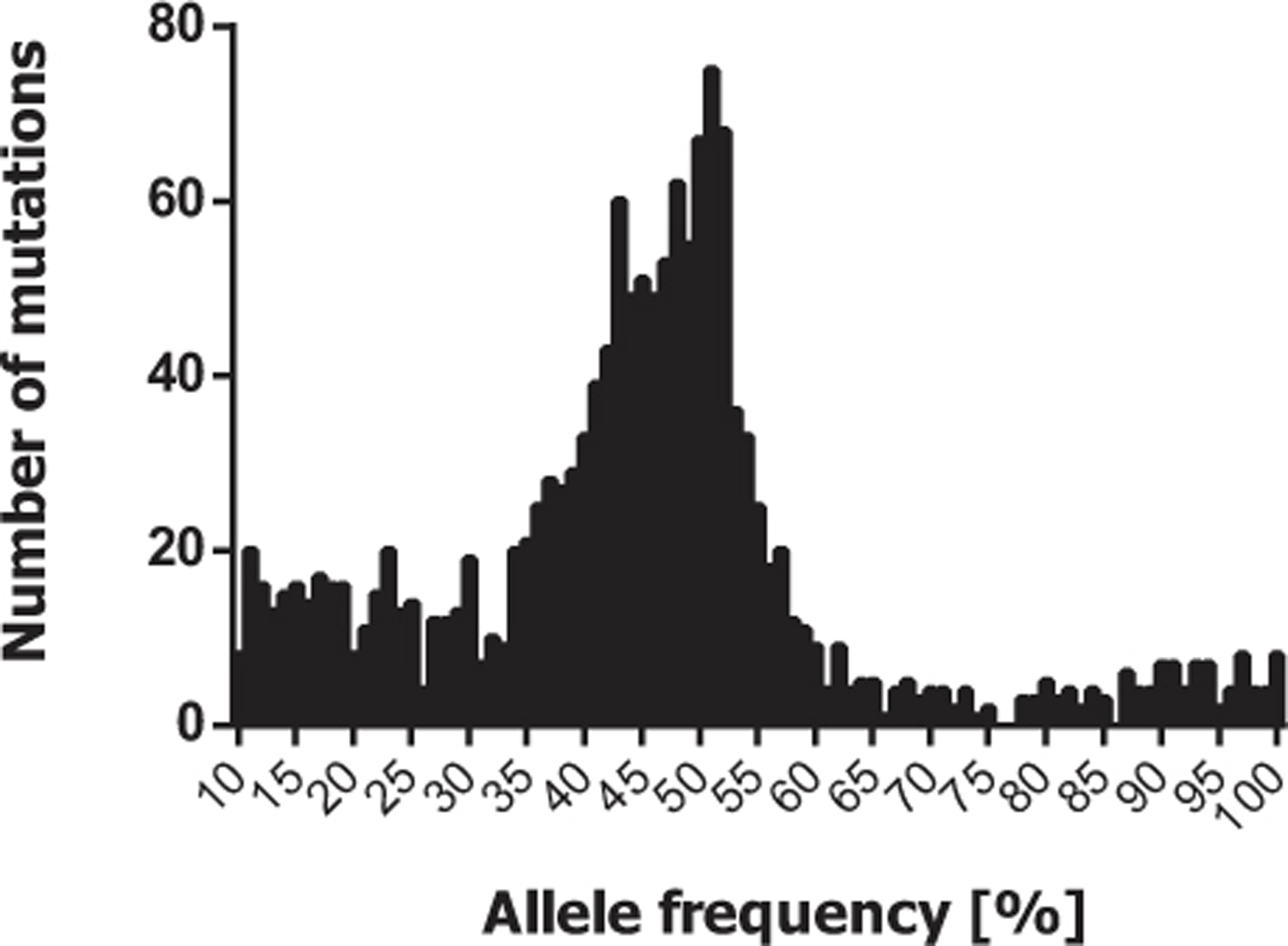

Supplement: Supplementary Figure 2 [file bcj20173x4.tif]

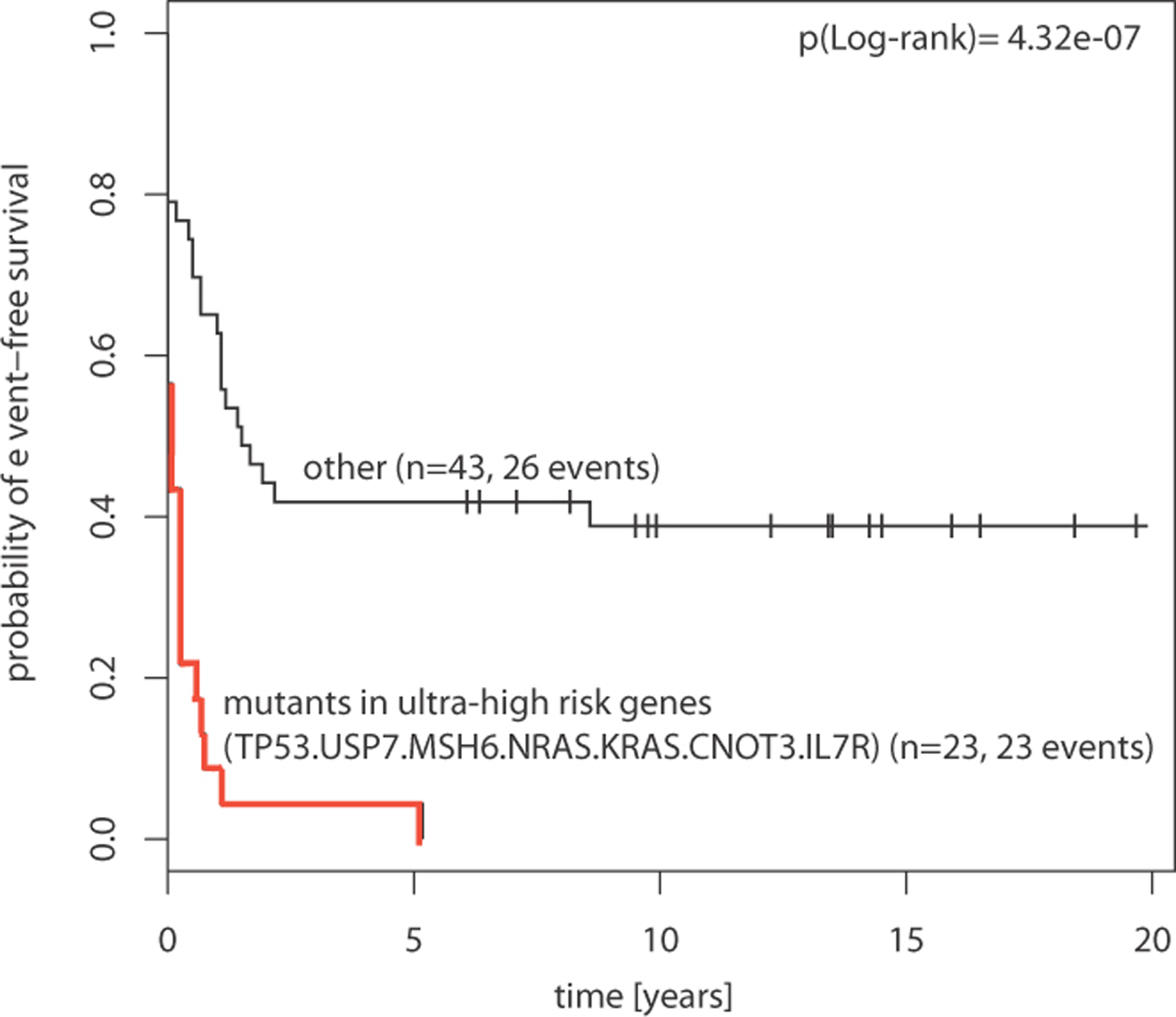

Supplement: Supplementary Figure 3 [file bcj20173x5.tif]

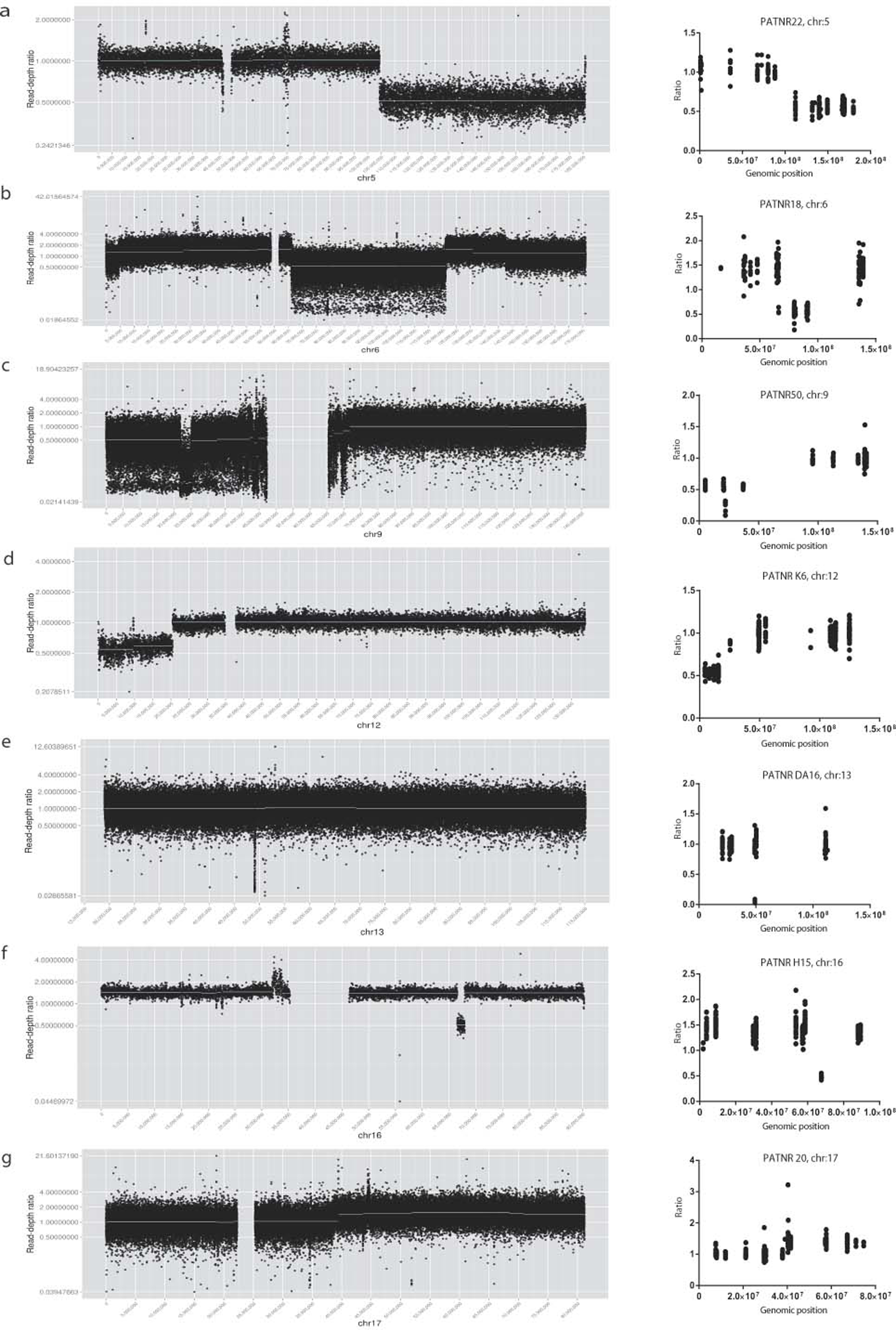

Supplement: Supplementary Figure 4 [file bcj20173x6.tif]
